# The uremic toxin *p*-cresyl sulfate induces proliferation and migration of clear cell renal cell carcinoma via microRNA-21/ HIF-1α axis signals

**DOI:** 10.1038/s41598-019-39646-9

**Published:** 2019-03-01

**Authors:** Tsai-Kun Wu, Chyou-Wei Wei, Ying-Ru Pan, Ren-Jun Hsu, Chung-Yi Wu, Yung-Luen Yu

**Affiliations:** 1 The Ph.D. Program for Cancer Biology and Drug Discovery, China Medical University and Academia Sinica, Taichung, 404 Taiwan; 2 Division of Renal Medicine, Tungs’ Taichung Metroharbor Hospital, Taichung, 435 Taiwan; 3 Deparment of Nutrition, Master Program of Biomedical Nutrition, Hungkuang University, Taichung, 433 Taiwan; 4 Department of Nursing, Hungkuang University, Taichung, 433 Taiwan; 5 Graduate Institute of Life Sciences, National Defense Medical Center, Taipei, 114 Taiwan; 6 The Genomics Research Center, Academia Sinica, Taipei, 115 Taiwan; 7 Graduate Institute of Biomedical Sciences, China Medical University, Taichung, 404 Taiwan; 8 Drug Development Center, China Medical University, Taichung, 404 Taiwan; 9 Center for Molecular Medicine, China Medical University Hospital, Taichung, 404 Taiwan; 10 Department of Biotechnology, Asia University, Taichung, 413 Taiwan

## Abstract

*p*-Cresyl sulfate (*p*CS), a uremic toxin, can cause renal damage and dysfunction. Studies suggest that renal dysfunction increases the prevalence of renal cancer. However, the effect of *p*CS on the proliferation and migration of renal cancer is unclear. Clear cell renal cell carcinoma (ccRCC) expresses mutant von Hippel-Lindau gene and is difficult to treat. Hypoxia-inducible factor-1α and 2-α (HIF-1α and HIF-2α) as well as microRNA-21 (miR-21) can regulate the proliferation and migration of ccRCC cells. However, the association between HIF-α and miR-21 in ccRCC remains unclear. Therefore, the effects of *p*CS on ccRCC cells were investigated for HIF-α and miR-21 signals. Our results showed that *p*CS induced overexpression of HIF-1α and promoted the proliferation and regulated epithelial-mesenchymal transition-related proteins, including E-cadherin, fibronectin, twist and vimentin in ccRCC cells. *p*CS treatment increased miR-21 expression. Specifically, inhibition of miR-21 blocked *p*CS-induced proliferation and migration. Taken together, the present results demonstrate that *p*CS directly induced the proliferation and migration of ccRCC cells through mechanisms involving miR-21/HIF-1α signaling pathways.

## Introduction

Clear cell renal cell carcinoma (ccRCC) is the most common subtype of renal cancers^1,2^. Clinically, approximately 70–80% of ccRCC is found in renal cancers^3^. Von Hippel-Lindau gene (VHL), a tumor suppressor gene, is found in various cancers^4^. VHL protein, a member of the E3-ubiquitin ligase complex, can bind to hypoxia-inducible factor-1α and 2-α (HIF-1α and HIF-2α) to cause HIF-α degradation^5^. A previous study showed that VHL inhibits cell proliferation by interacting with HIF^6^. However, in most ccRCC cases, VHL is mutated and deficient^7,8^. Currently, nephrectomy is the major treatment for ccRCC^9,10^; as ccRCC is not sensitive to various drugs, chemotherapy is not an effective treatment^11,12^. Therefore, it is important to understand the mechanisms of the proliferation and migration of ccRCC to develop new therapies.

Indoxyl sulfate (IS) and *p*-cresyl sulfate (*p*CS) are 2 types of uremic toxins metabolized from tryptophan and tyrosine, respectively, in the intestine and can cause renal dysfunction. Tyrosine is converted into *p*-cresol through a series of reactions by microbiota in the distal colon, such as deamination, transamination, and decarboxylation. *p*-Cresol is further detoxified into *p*CS in the mucosa of the colon and in the liver. In circulation, *p*CS mostly binds to albumin and is excreted in the kidney. The free fraction of *p*CS is filtered at the glomerulus and the protein-bound fraction is secreted at the  tubular epithelial cells^13^. The gut microbiota is also altered in patients with chronic kidney disease (CKD) with changes in the composition of microbiota and overgrowth of bacteria in the small intestine and colon, resulting in higher levels of *p*-cresol^14^. In CKD patients, excretion is impaired; in patients with hemodialysis, protein-bound *p*CS is difficult remove by dialysis. *p*CS accumulates as renal function deteriorates, with 100–200 μM as the mean serum total levels of *p*CS observed in uremic patients^15^. Accumulation of *p*CS causes renal dysfunction and disease progression by inducing oxidative damage and endothelial dysfunction^16,17^. Previous studies found that renal dysfunction is related to the risk of renal cell carcinoma, particularly ccRCC^18,19^. *p*CS can induce proliferation and migration of rat aortic vascular smooth muscle cells^20^ and epithelial-mesenchymal transition (EMT) in kidney fibrosis^21^. Based on these studies, we predicted that *p*CS influences proliferation, EMT, and migration in ccRCC. Therefore, the aim of this study was to determine the effect of *p*CS on the proliferation and migration of ccRCC cells and the related mechanisms.

HIF-1α and HIF-2α are transcription factors^22,23^. Most patients with ccRCC have high expression of HIF-1α^24^, and studies have shown that HIF-1α plays an important role in the proliferation of ccRCC^10,24,25^ and regulates EMT in ccRCC and tubular epithelial cells^26,27^. Fibronectin, twist, vimentin, and E-cadherin are associated with EMT and are required for cell migration^28,29^. Additionally, inhibition of HIF-1α can reduce the migration of ccRCC cells^30^. These results suggest that HIF-1α plays an important role in the proliferation, EMT, and cell migration of ccRCC. HIF-2α also regulates cell proliferation and migration in ccRCC^7,31^. Therefore, the expression of HIF-α, EMT-related proteins, and migration were investigated in *p*CS-treated ccRCC cells in this study.

MicroRNAs, which are 19–25 nucleotides in length, can influence gene expression^32^. MicroRNAs can interact with the 3′-untranslated regions of mRNAs to cause mRNA degradation and inhibit translation^33^. MicroRNA-21 (miR-21) is highly expressed in various cancers^34^. Studies have indicated that miR-21 can induce cell proliferation and EMT in ccRCC^35,36^. MiR-21 can activate HIF-1α expression in various cells including prostate cancer, retinal pigment epithelia, cervical cancer, and human stem cells^37–39^, but its role in ccRCC remains unclear. Therefore, the miR-21/HIF-1α axis signals were studied in *p*CS-treated ccRCC cells to examine cell proliferation and migration.

## Results

### Effect of *p*CS on cell proliferation in dose- and time-dependent manner

**Figure 1:**
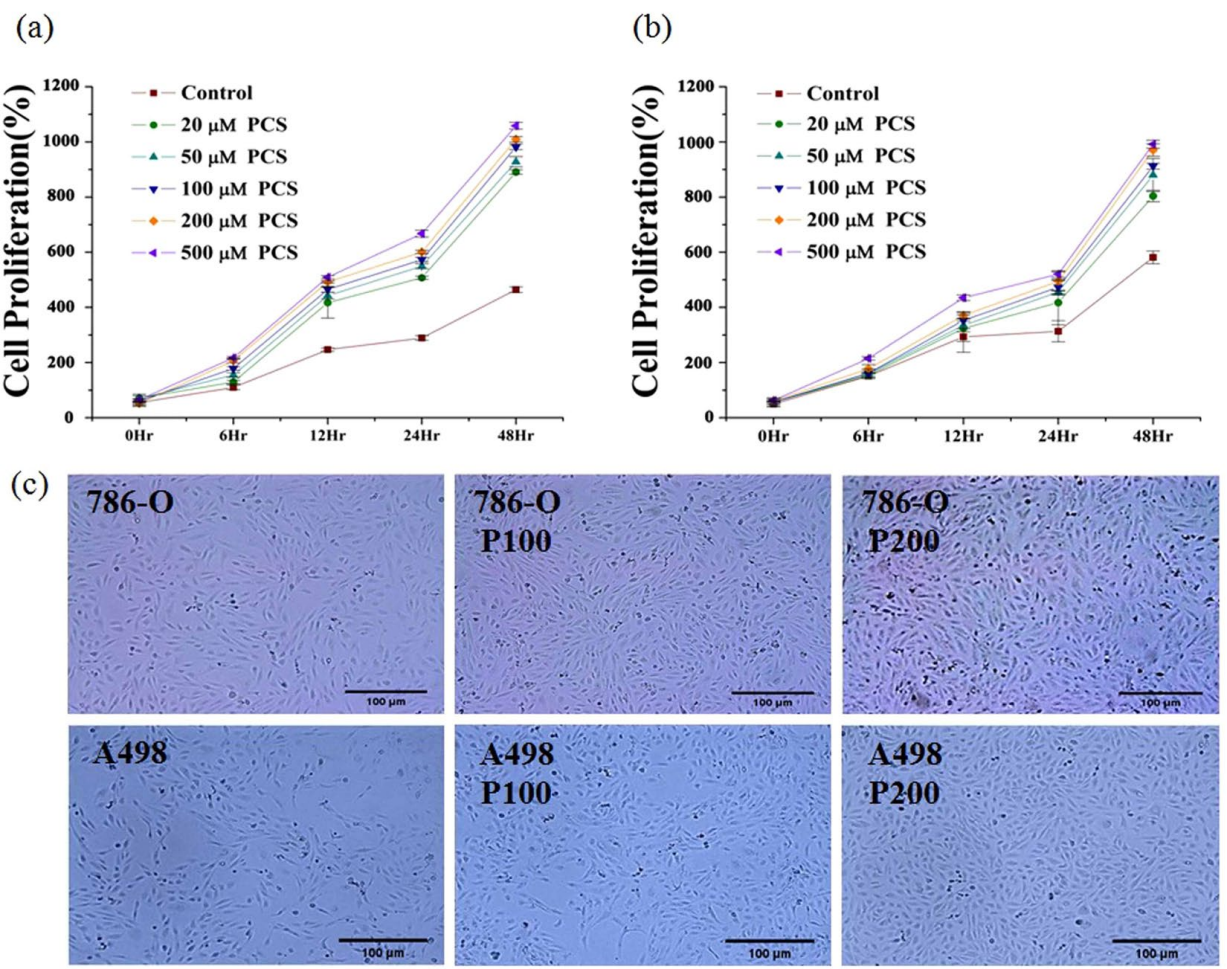
Effect of *p*-cresyl sulfate (*p*CS) on proliferation of clear cell renal cell carcinoma (ccRCC) was time-dependent. Various concentrations of *p*CS (20–500 µM) were tested on 786-O (**a**) and A498 (**b**) cells. Cells were assayed for 48 h (**c**) after treatment with 100 and 200 μM *p*CS or none (P100, P200, or control). Cell proliferation was measured by WST1 assay as cell optical density at 48 h. Values are represented as the mean ± standard deviation from three independent experiments.

Cells from two cell lines, ccRCC 786-O and A498, were treated with 20, 50, 100, 200, and 500 μM of *p*CS for 48 h. Cell proliferation was measured by the WST-1 assay and the optical density of the cell culture was determined. As shown in Fig. 1, cell proliferation by *p*CS treatment of both cells was increased significantly at 12 h for 786-O cells and 24 h for A498 cells. *p*CS-induced cell proliferation was time-dependent in both 786-O and A498 cells (Fig. 1a,b). Cells treated with control, 100 or 200 μM *p*CS for 48 h are shown in Fig. 1c.

### Effect of *p*CS on HIF-1α, HIF-2α and VHL levels

**Figure 2:**
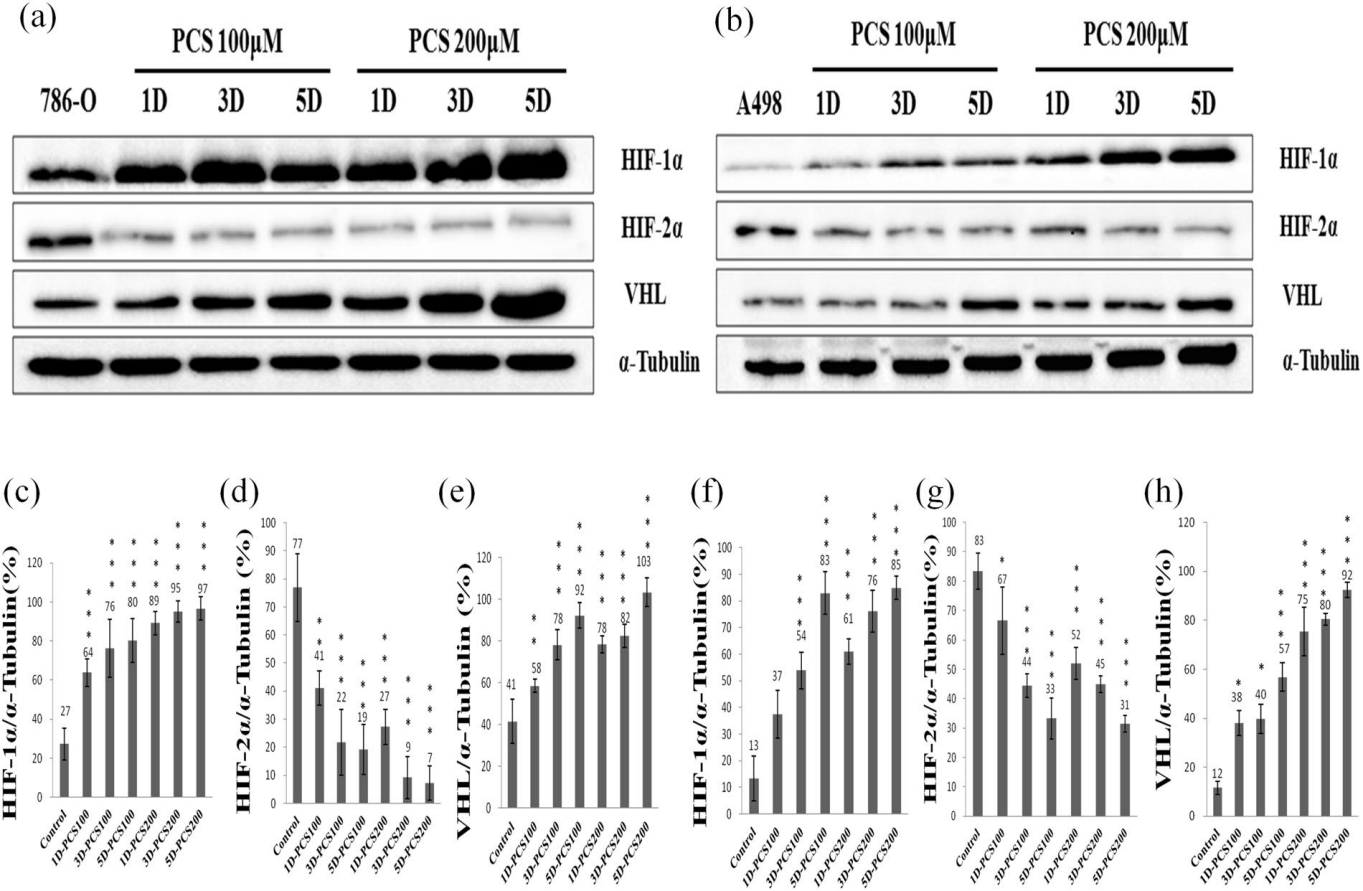
Effect of *p*CS on HIF-1α, HIF-2α, and VHL levels of 786-O and A498 cells. Cells treated with 100 or 200 μM *p*CS for 1 day (1D), 3 days (3D), or 5 days (5D) and indicated as 1D-PCS100 or 5D-PCS200. Protein expression in 786-O (**a**) and A498 (**b**) cells was assayed by Western blotting. The ratios of HIF-1α, HIF-2α, and VHL to α-tubulin were compared in 786-O cells (**c**–**e**) and A498 cells (**f**–**h**), respectively. HIF-1α and VHL levels were increased and HIF-2α level decreased in both *p*CS-treated cells as compared to the control. (*P < 0.05, **P < 0.01 and ***P < 0.001, respectively).

HIF-1α and HIF-2α transcription factors can regulate the cell proliferation of renal cancer^5^. We investigated whether *p*CS-induced proliferation was related to HIF-1α and HIF-2α. 786-O and A498 cells were treated with 100 or 200 μM *p*CS for 5 days. HIF-1α, HIF-2α, and VHL levels and the ratios of their expressions are shown in Fig. 2. *p*CS-induced cell proliferation of 786-O cells was related to HIF-1α signals and decreased levels of HIF-2α (Fig. 2a–d). Similarly, HIF-1α was increased and HIF-2α was decreased in *p*CS-treated A498 cells (Fig. 2b,f,g). Interestingly, VHL levels were also increased in both *p*CS-treated 786-O (Fig. 2a,e) and A498 cells (Fig. 2b,h). This result suggests that HIF-1α signals play an important role in *p*CS-induced proliferation of ccRCC cells.

### Effect of *p*CS on EMT and cell migration

**Figure 3:**
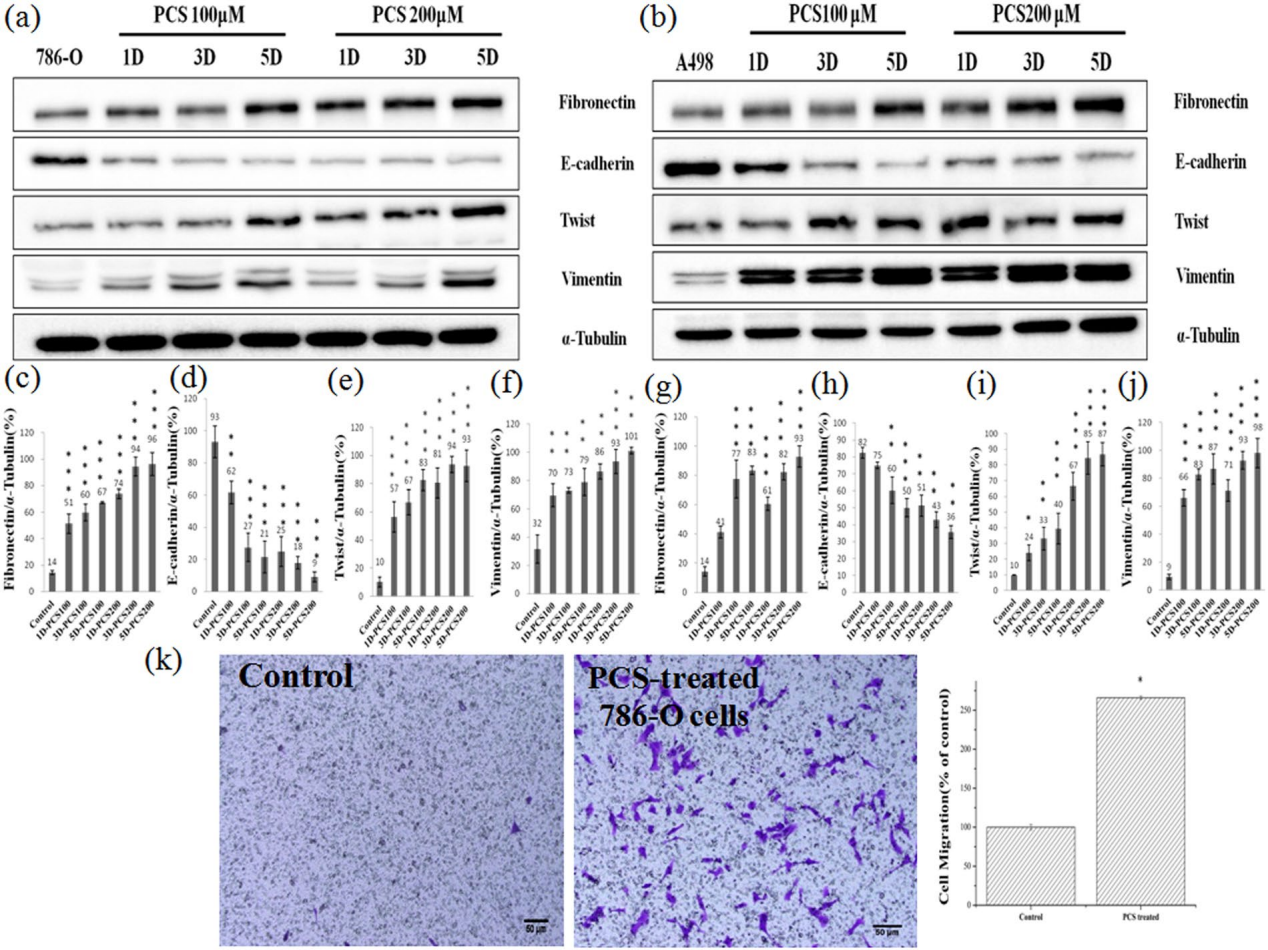
Effect of *p*CS on expression of EMT-related proteins in 786-O (**a**) and A498 (**b**) cells. Western blotting was used and ratios of fibronectin, E-cadherin, twist, and vimentin to α-tubulin were compared in 786-O cells (**c**–**f**) and A498 cells (**g**–**j**), respectively. The migration and percentage (**k**) were determined for 786-O cells using a Transwell system with and without 100 μM *p*CS treatment. Fibronectin, twist, and vimentin expression was increased, while E-cadherin was decreased in *p*CS-treated 786-O and A498 cells, indicating that *p*CS induced EMT in ccRCC cells. *P < 0.05, **P < 0.01 and ***P < 0.001, as compared to the control.

Because HIF-1α regulates EMT-related proteins^29^, we studied the effect of *p*CS on EMT in ccRCC cells. EMT-related proteins (including fibronectin, twist, vimentin, and E-cadherin) were determined by Western blotting in *p*CS-treated 786-O and A498 cells (Fig. 3a,b). The results showed that fibronectin, twist, and vimentin expression was increased, while E-cadherin was decreased in *p*CS-treated 786-O and A498 cells (Fig. 3) and indicated that *p*CS induced EMT in ccRCC cells. The effect of *p*CS on cell migration was further investigated in ccRCC cells. As shown in Fig. 3k, *p*CS promoted the migration of 786-O cells as compared to the control. The relative density of migration is shown in Fig. 3k. Similarly, *p*CS promoted the migration of A498 cells (data not shown). These results indicate that *p*CS induces EMT and migration of ccRCC cells.

### Effect of HIF-1α knockdown on *p*CS-induced proliferation, EMT and migration

**Figure 4:**
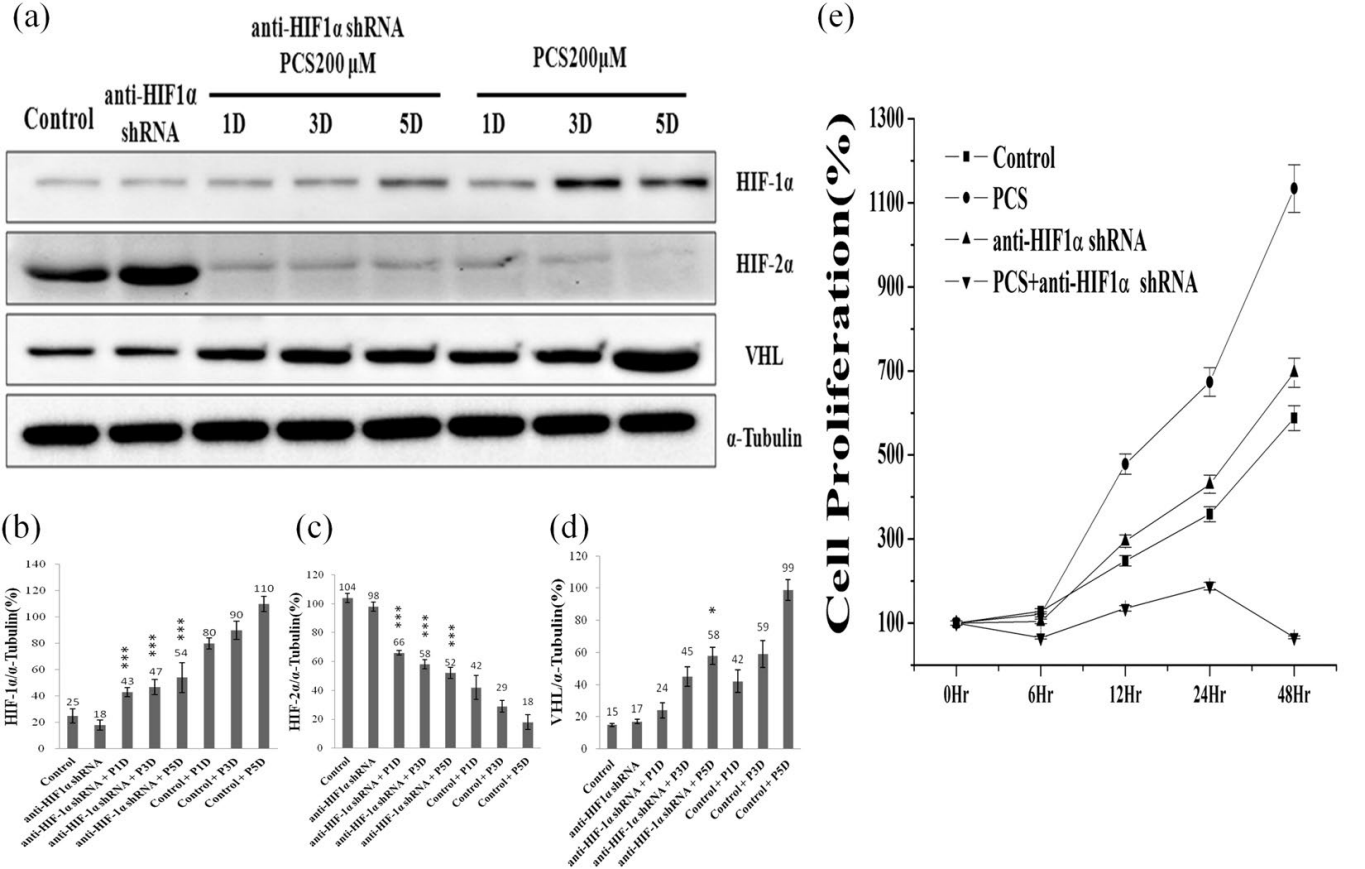
Effect of HIF-1α knockdown on HIF-1α, HIF-2α, and VHL expression in 786-O cells. HIF-1α, HIF-2α, VHL, and α-tubulin levels were assayed by Western blotting (**a**) with or without shRNA HIF1α in 200 μM *p*CS-treated cells at day(s) 1–5. Ratios of HIF-1α (**b**), HIF-2α (**c**), and VHL (**d**) to α-tubulin were compared. Knockdown of HIF-1α promoted the expression of HIF-2α at day(s) 1–5 and inhibited VHL expression only at day 5. HIF-1α knockdown inhibits *p*CS-induced proliferation (**e**). Values were represented as the mean ± standard deviation from three independent experiments. *P < 0.05 and ***P < 0.001, as compared to the control group. *p*CS-treated day(s) 1, 3, and 5 are indicated as P1D, P3D, and P5D, respectively. Anti-HIF-1α shRNA indicated as HIF-1α knockdown with shRNA.

**Figure 5:**
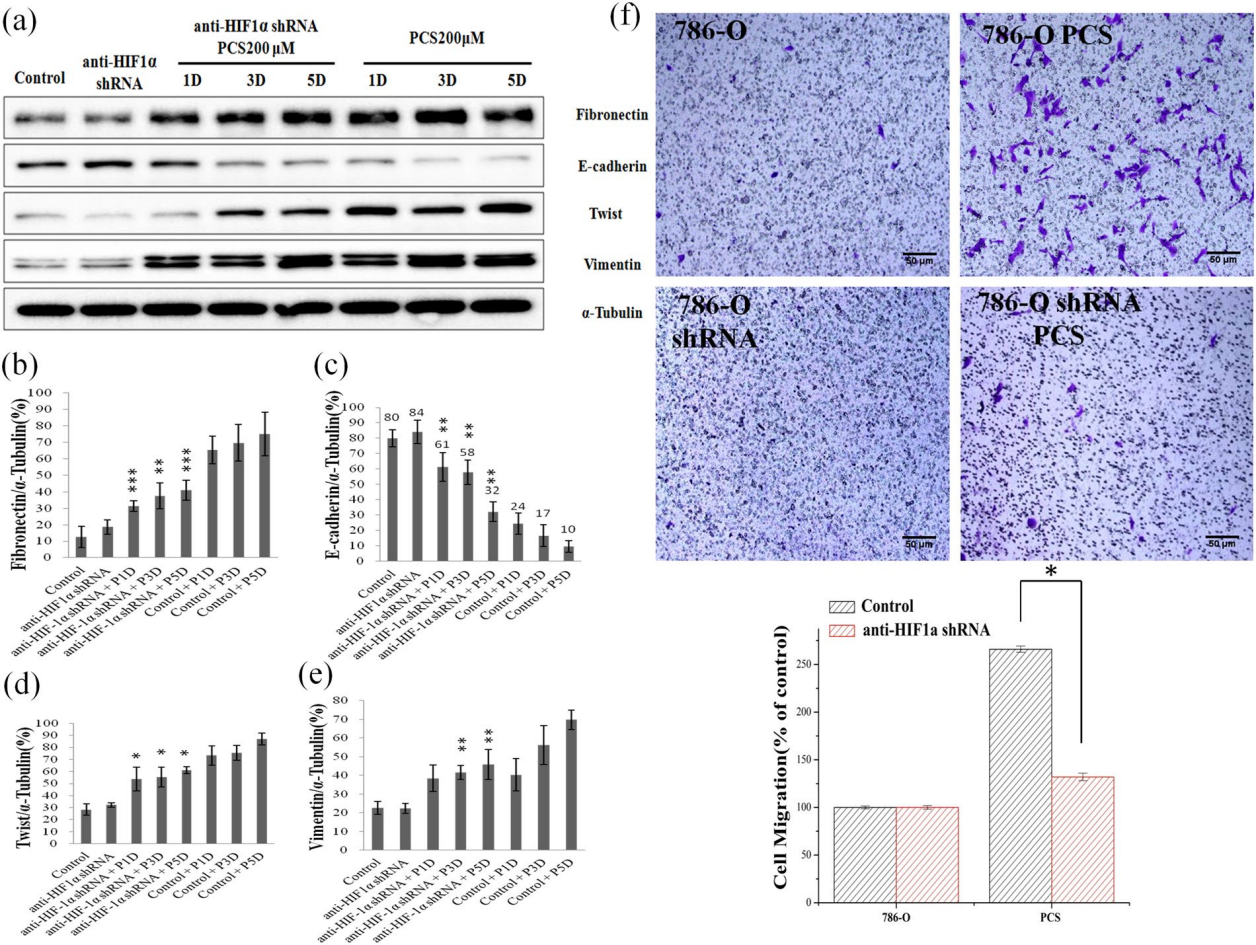
Effect of HIF-1α knockdown on expression of EMT-related proteins in 786-O cells. Protein expression was assayed by Western blotting (**a**) with or without shHIF1α in 200 μM *p*CS-treated cells at day(s) 1–5. Ratios of fibronectin (**b**), E-cadherin (**c**), twist (**d**), and vimentin (**e**) to α-tubulin were compared. HIF-1α knockdown inhibited *p*CS-induced cell migration (**f**). *p*CS-induced cell migration was inhibited by knockdown of HIF-1α. *P < 0.05, **P < 0.01, and ***P < 0.001 compared to the control. Anti-HIF-1α shRNA indicated as HIF-1α knockdown with shRNA. *p*CS-treated day(s) 1, 3, and 5 are indicated as P1D, P3D, and P5D, respectively. 786-O shRNA cells: HIF-1α knockdown 786-O cells and 786-O shRNA PCS: 786-O shRNA cells treated with *p*CS.

To determine whether *p*CS induces EMT, migration, and proliferation via HIF-1α, HIF-1α was knocked down with an anti-HIF-1α short hairpin RNA (shRNA). Knockdown of HIF-1α promoted the expression of HIF-2α at days 1, 3, and 5 (Fig. 4a–c). However, knockdown of HIF-1α significantly inhibited VHL expression in *p*CS-treated 786-O cells only at day 5 (Fig. 4a,d). Additionally, knockdown of HIF-1α inhibited the expression of fibronectin, twist, and vimentin and promoted the expression of E-cadherin (Fig. 5a). *p*CS-induced cell migration (Figs 3k and 5f) was inhibited by knockdown of HIF-1α (Fig. 5f). Knockdown of HIF-1α also inhibited *p*CS-induced proliferation of 786-O cells (Fig. 4e). Similar results were observed for *p*CS-treated A498 cells (data not shown). Overall, these results suggest *p*CS induced EMT, migration, and proliferation via HIF-1α signals.

### miR-21 mediates *p*CS-induced EMT and proliferation of ccRCC

**Figure 6:**
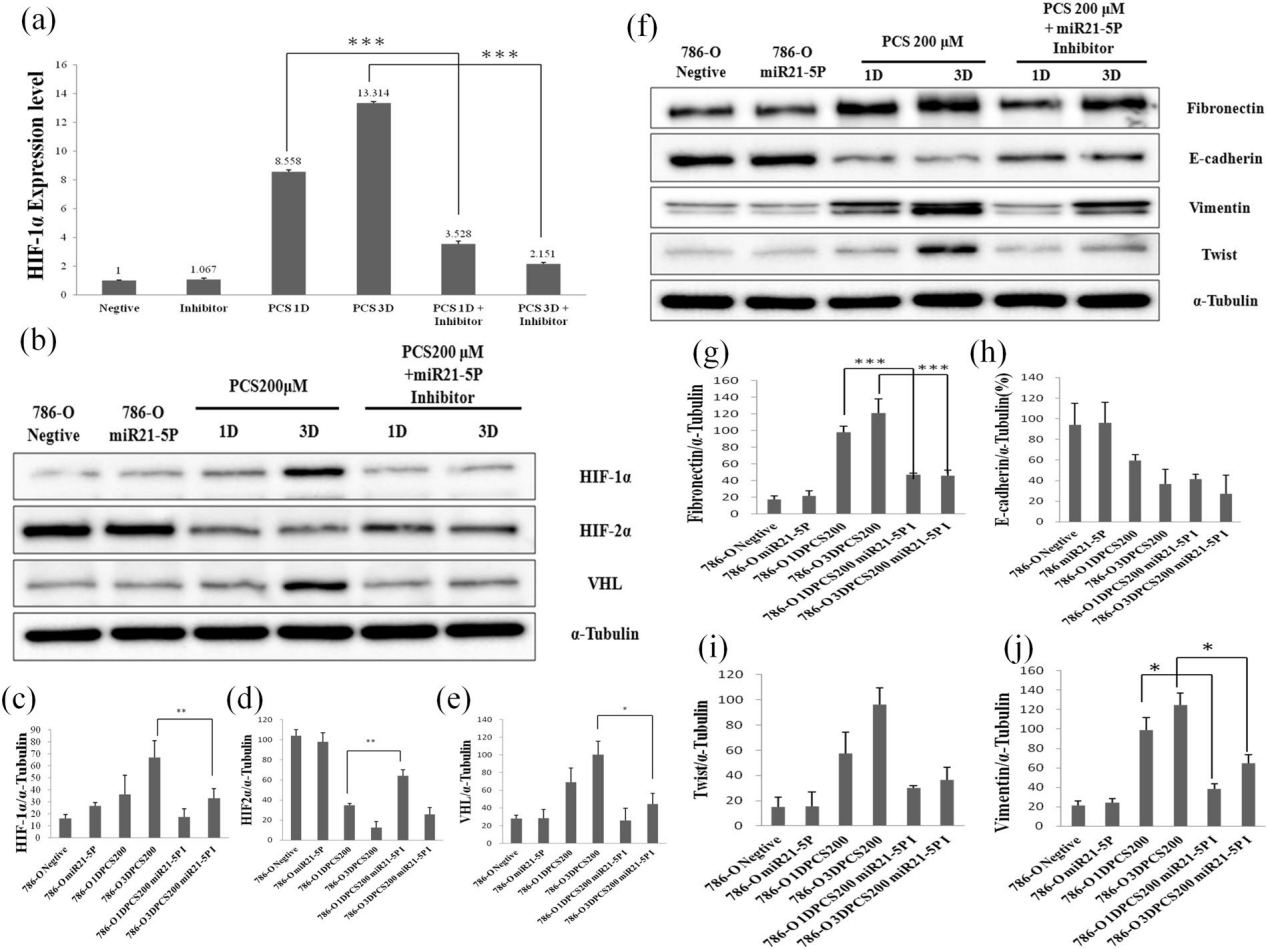
Effect of miR-21 inhibitor on *p*CS-induced HIF-1α mRNA expression by PCR (**a**) and HIF-1α, HIF2α, and VHL expression by western blotting (**b**). HIF-1α levels in 786-O cells were compared with miR-21 inhibitor-treated group (miR21-5P), *p*CS-treated-1 and 3 day groups (1D and 3DPCS200), as well as *p*CS plus miR-21 inhibitor-treated-1 and 3 day groups (1DPCS200 miR21-5PI and 3DPCS200 miR21-5PI). Ratios of HIF-1α (**c**), HIF-2α (**d**), and VHL (**e**) to α-tubulin were compared. miR-21 inhibition regulated *p*CS-induced expression of EMT-related proteins (**f**). Ratios of fibronectin (**g**), E-cadherin (**h**), twist (**i**), and vimentin (**j**) to α-tubulin were also compared. The inhibitor of miR-21 significantly inhibited the expression of HIF-1α, fibronectin and vimentin in *p*CS-treated cells, suggesting that *p*CS increases miR-21 to cause overexpression of HIF-1α and EMT in 786-O cells. Values are expressed as the mean ± standard deviation from three independent experiments. *P < 0.05, **P < 0.01, and ***P < 0.001, compared to the *p*CS-treated group.

Previous studies demonstrated that miR-21 promotes the proliferation and EMT of ccRCC cells^38,39^. We examined the role of miR-21 in *p*CS-induced proliferation and EMT-mediated protein expression. The results showed that the miR21 inhibitor reduced the expression of HIF-1α mRNA at days 1 and 3 (Fig. 6a). Additionally, overexpression of HIF-1α and VHL proteins was inhibited and expression of HIF-2α was promoted in *p*CS-treated cells with miR-21 inhibitor(Fig. 6b). The inhibitor of miR-21 also significantly inhibited the expression of fibronectin and vimentin in *p*CS-treated cells (Fig. 6g,j). Overall, our results suggest that *p*CS increases miR-21 to cause cell proliferation and EMT in ccRCC cells.

## Discussion

Previous reports showed that patients with end-stage renal disease (ESRD) generally have high levels of uremic toxins containing IS and *p*CS^40,41^. Clinical cases showed that patients with ccRCC are closely associated with ESRD^42,43^. However, direct evidence linking *p*CS and ccRCC progression is lacking. Our results demonstrated that *p*CS induced the proliferation and migration of ccRCC cells and suggested that the progression of ccRCC is related to *p*CS in the kidneys of patients. Therefore, removing *p*CS may prevent ccRCC progression in ESRD patients. AST-120, an approved clinical drug, decreases *p*CS levels^44,45^ and may be useful for preventing ccRCC progression, but further studies are needed to confirm this aspect. Other methods are also available for decreasing *p*CS such as renal replacement therapy of hemodiafiltration, dietary intervention, laxative, and pro-, pre-, and syn-biotics^13,14^.

Accumulation of *p*CS increases oxidative stress in endothelial cells, reactive oxygen species in cardiomyocytes, and expression of DNA methyltransferase in HK2 cells^13^. A study showed that *p*CS activated the renin angiotensin aldosterone system and induced EMT^21^. Some studies showed that accumulation of reactive oxygen species leads to stabilization of HIF-1α^46,47^. Another study showed that the angiotensin system increased HIF-1α signals^48^.

HIF-1α and HIF-2α have similar structures containing transcription and oxygen-dependent degradation domains, but have different functions^49^. Additionally, both HIF-1α and HIF-2α can regulate cell proliferation and migration^7,24,25,28,31^. Some studies indicated that HIF1α acts as a renal tumor suppressor and HIF2α as a renal tumor inducer^50,51^. However, another study showed that HIF-1α inhibits and HIF-2α induces apoptosis^52^. Nevertheless, both HIF-1α and HIF-2α induce the proliferation of ccRCC cells^7,10,25,53^. Our results showed that *p*CS induced HIF-1α overexpression and promoted the proliferation of ccRCC cells. This suggests that HIF-1α plays an important role in promoting the proliferation of ccRCC cells. Our results on *p*CS-induced HIF-1α and reduced HIF-2α expression agreed with those of previous studies^54,55^.

VHL can interact with HIF-α to promote the degradation of HIF-α via the ubiquitin system, leading to blockage of HIF-α signals^56,57^. However, mutant VHL genes are found in most ccRCC cells, including in 786-O and A498 cells^58^. A study indicated that mutant VHL does not regulate HIF-α signals^52^. Our results showed that *p*CS induced HIF-1α and VHL and suppressed HIF-2α expression. We found that overexpression of mutant VHL in *p*CS-treated 786-O and A498 cells did not inhibit HIF-1α-induced proliferation. This may be explained by the data showing that overexpression of mutant VHL in ccRCC prevented UV-induced apoptosis^59^. Although the mechanism of *p*CS-induced overexpression of VHL remains unclear, *p*CS-induced overexpression of VHL may decrease apoptosis and lead to the progression of ccRCC.

**Figure 7:**
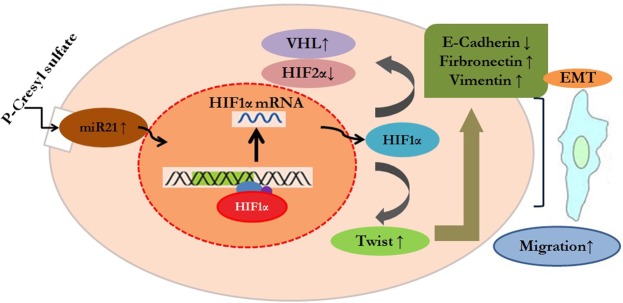
Hypothetical mechanism of *p*CS-induced proliferation and EMT of ccRCC. *p*CS treatment increased miR-21 expression in ccRCC and further induced overexpression of HIF-1α. HIF-1α promoted proliferation and regulated epithelial-mesenchymal transition (EMT)-related proteins including E-cadherin, fibronectin, twist, and vimentin to promote migration of ccRCC cells.

miR-21 in various cancers can activate HIF-1α signals to promote cell proliferation^37,38^. Both miR-21 and HIF-1α induced proliferation and EMT of ccRCC cells^25,28,35,36^, but it was unclear whether miR-21 upregulates HIF-1α in ccRCC. Our results showed that inhibition of miR-21 decreased HIF-1α expression in *p*CS-treated ccRCC cells. Therefore, miR-21 functions upstream of HIF-1α to regulate the proliferation and EMT of ccRCC cells. A hypothetical mechanism is shown in Fig. 7 and summarizes our study results. Briefly, *p*CS induced overexpression of miR-21, resulting in increased expression of HIF-1α and VHL and decreased HIF-2α. HIF-1α further promote the proliferation and migration of ccRCC cells. VHL is commonly mutated in ccRCC, and there are no reports regarding the relationship between miR-21 and VHL in ccRCC. However, a previous study indicated miR-21 can target VHL to regulate cell growth and that miR-21 inhibition induces VHL expression and inhibits the proliferation of glioblastomas^60^. In contrast, our results showed that inhibition of miR-21 decreased the expression of VHL and inhibited *p*CS-induced proliferation in ccRCC cells. Because wild-type VHL in glioblastoma inhibits cell growth while mutant VHL in ccRCC inhibits apoptosis, miR-21 has distinct effects on VHL expression and regulates cell growth in different cells.

In summary, we demonstrated that *p*CS-induced proliferation and EMT of ccRCC was mainly mediated by miR-21/HIF-1α signals.

## Materials and Methods

### Materials

*p*-Cresyl sulfate (*p*CS) was obtained from Sigma (Sigma-Aldrich, St. Louis, MO, USA). The Millicell cell culture chamber was obtained from Millipore (Millipore, Billerica, MA, USA). Rabbit polyclonal HIF1α antibody (1:1000) was obtained from Genetex (Alton Parkway, CA, USA). Rabbit monoclonal HIF2α antibody (1:1000), rabbit polyclonal VHL antibody (1:1000), rabbit monoclonal E-cadherin antibody (1:1000), rabbit monoclonal vimentin antibody (1:1000), and rabbit polyclonal α-tubulin antibody (1:5000) were from Cell Signaling Technology (Danvers, MA, USA). Rabbit polyclonal fibronectin antibody (1:2000) and mouse monoclonal twist antibody (1:500) were from Abcam (Cambridge, UK). Secondary antibodies (anti-mouse IgG horseradish peroxidase-linked and anti-rabbit IgG horseradish peroxidase-linked) were form Cell Signaling Technology. Primers were synthesize by Integrated DNA Technologies (Coralville, IA, USA).

### Cell culture

A498 and 786-O cells of ccRCC were kindly provided by Dr. Ren-Jun Hsu (Graduate Institute of Life Sciences, National Defense Medical Center, Taipei, Taiwan). Both cells were cultured with RPMI 1640 medium (HyClone, Logan, UT, USA) supplemented with 10% fetal bovine serum (Invirogen, Carlsbad, CA, USA), 2 mM l-glutamine, 1.5 g/L sodium bicarbonate, 4.5 g/L glucose, 10 mM HEPES, and 1.0 mM sodium pyruvate. The cells were maintained in a humidified atmosphere containing 5% CO_2_ at 37 °C.

### Cell proliferation assay

Cell proliferation was measured using a WST-1 assay kit (BioVision, Milpitas, CA, USA). The cells were cultured in 96-well culture plates (8 × 10^3^ cells/well). WST-1 reagent was added to the wells of the experimental and control groups at 6, 12, 24, and 48 h. After incubation for 3 h (37 °C, 5% CO_2_), an aliquot of culture supernatant was measured at 450 nm with a Multiskan™ FC Microplate Photometer (Molecular Devices, Sunnyvale, CA, USA). The absorbance of WST-1 reagent without cells was used as the blank.

### SDS-PAGE and western blotting

The cells were collected and washed twice with PBS (137 mM NaCl, 2.7 mM KCl, 10 mM Na_2_HPO_4_, 2 mM KH_2_PO_4_), and then lysed with NETN buffer (20 mM Tris at pH 8.0, 150 mM NaCl, 1 mM EDTA at pH 8.0, 0.5% Nonidet P-40) plus protease and phosphatase inhibitors (25 mM NaF, 2 mM Na_3_VO_4_, 0.1 mM PMSF, 20 μg/mL aprotinin) and sonicated with a sonicator (ChromTech UP-800). After centrifugation at 15,000 × *g* for 15 min at 4 °C, the soluble extraction containing proteins was collected from the supernatant. The protein concentration was measured with a protein assay kit (Thermo Fischer Scientific, Waltham, MA, USA). Equal quantities (approximately 40 μg) of samples were loaded onto 6%, 10%, and 15% SDS-PAGE gel and separated at a voltage of 100V. Proteins in the SDS-PAGE gel were then transferred to a polyvinylidene fluoride membrane (Millipore). The membrane was treated with 5% skim milk in TBST buffer (TBS containing 0.1% Tween-20) for 1 h at 26.5 °C and then hybridized with primary antibody at 4 °C with gentle agitation overnight. After washing with TBST three times, the membrane was incubated with secondary antibody for 1 h at 26.5 °C. The protein was detected by using the enhanced chemiluminescence detection reagent (GE Healthcare, Little Chalfont, UK) and observed with a Luminescence Image Analysis system (LAS-4000, GE Healthcare). The protein levels were quantified by using Image J software (NIH, Bethesda, MD, USA) and protein percentage was indicated as target protein level/tubulin protein level × 100%.

### Cell migration assay

Cell migration was determined by using Millicell cell culture chambers (24-well, 8-μm chambers, Millipore) according to the manufacturer’s instructions. Briefly, the Matrigel was re-hydrated with RPMI 1640 media (1:4) immediately for 1 h before the migration assay. Cells (5 × 10^4^) were suspended in 200 μL serum-free medium then added to the upper chamber of Matrigel-coated filter inserts. After treatment with surfactin, 700 μL RPMI 1640 (containing 10% fetal bovine serum) was added to the bottom well as a chemoattractant. Next, the chambers were incubated for 24 h. Migrated cells attached to the lower surface of the filter. The cells were fixed and stained with 2% ethanol containing 0.2% crystal violet. Migrated cells were counted under a light microscope (40x) (OLYMPUS, IX-71, Tokyo, Japan) and absorbance was measured at 470 nm. The migration percentage was indicated as A470 experimental group/A470 control group × 100%.

### Knockdown of HIF-1α

HIF-1α knockdown was performed with specific short hairpin RNAs (shRNAs) delivered by a lentivirus system from the National RNAi Core Facility (Academia Sinica, Taipei, Taiwan) according to the protocol. Control shRNA were produced by using 2.5 μg pLKO.1-Luc, 0.25 μg pMDG, and 2.25 μg pCMV-ΔR8.91 plasmids cotransfected into 293 T cells with Lipofectamine agent (Invitrogen, Carlsbad, CA, USA). Anti-HIF-1α shRNA was produced by using 2.5 μg pLKO.1-HIF-1α, 25 μg pMDG, and 2.25 μg pCMV-ΔR8.91 plasmids cotransfected into 293 T cells with Lipofectamine agent. After 6 h, the medium was replaced with RPMI 1640 containing 1% bovine serum albumin for 24 h. The lentiviral particles with control shRNA or anti-HIF-1α shRNA were collected using a 0.22-μM filter and then stored at −80 °C. For gene knockdown, cells were transduced with the lentiviral particles with 8 μg/mL polybrene. After 24 h, 3 μg/mL puromycin was added to the culture medium and selected for 3 days.

### Inhibition of miR-21

Cells were cultured to 50–60% confluence and transfected with a miR-21-5P inhibitor and negative control miRNA inhibitor (Integrated DNA Technologies) by using siLenFect^TM^ lipid reagent (Bio-Rad, Hercules, CA, USA) in serum-free Opti-MEM medium according to the manufacturer’s instructions. The final concentration of the oligomers was 25 nM. After transfection for 24 h, the medium was replaced with fresh RPMI medium containing 10% fetal bovine serum. The levels of miR-21 were analyzed by quantitative real-time polymerase chain reaction (qRT-PCR).

### Determination of RNA expression levels

The RNA expression levels of miR-21, HIF1α and were determined by qRT-PCR. The optimized PCR assay of 20 μL PCR volume contained 10 µL of iTaq Universal Probes Supermix, 2 μL of TaqMan Gene Expression Assay, and water to a volume of 20 μL. All reagents were mixed and distributed into a 96-well PCR plate before adding 2 µL of cDNA (1–100 ng). The PCR program was as follows: 95 °C for 30 s, followed by 40 cycles at 95 °C for 1 s and 60 °C for 60 s, during which fluorescence data were collected. Total RNA was extracted using the Purezol kit (Bio-Rad) according to the manufacturer’s protocol. Next, 1 μg of total RNA was used to synthesize cDNA with a cDNA Synthesis kit (Bio-Rad). The expression levels of B2M and HIF1α were quantified by qRT-PCR using the iTaq Universal probe Supermix kit (Bio-Rad) and StepOne plus Real-time PCR system (Applied Biosystems, Foster City, CA, USA). Primers used in this experiment were as follows: HIF1α: 5′-CAACCCAGACA- TATCCACCTC-3′ (forward (F)), 5′-CTCTGATCATCTGACCAAAACTCTA-3′ (reverse (R)). The relative expression level of each gene was calculated by using the 2^−ΔΔCt^ method). All data were obtained from three independent experiments.

### Statistical analysis

Data are presented as the mean ± SE from at least three independent experiments. One-way analysis of variance was used to compare the experimental data. Two-way analysis of variance was used to compare data obtained from different treatment concentrations and incubation times. The data were analyzed with SPSS Statistics v18.0 (SPSS, Inc., Chicago, IL, USA). A P value < 0.05 was considered statistically significant.

## Supplementary information

Supplementary Information

## References

[1] Wang D (2017). MicroRNA-30e-3p inhibits cell invasion and migration in clear cell renal cell carcinoma by targeting Snail1. Oncol Lett.

[2] Liu, N. *et al*. Percutaneous radiofrequency ablation for renal cell carcinoma vs. partial nephrectomy: Comparison of long-term oncologic outcomes in both clear cell and non-clear cell of the most common subtype. *Urol Oncol*, 10.1016/j.urolonc.2017.03.014 (2017). 10.1016/j.urolonc.2017.03.014 28408296

[3] Jin P, Wang J, Liu Y (2017). Downregulation of a novel long non-coding RNA, LOC389332, is associated with poor prognosis and tumor progression in clear cell renal cell carcinoma. Exp Ther Med.

[4] Yang L (2016). The Clinicopathological Significance of Epigenetic Silencing of VHL Promoter and Renal Cell Carcinoma: A Meta-Analysis. Cell Physiol Biochem.

[5] Keith B, Johnson RS, Simon MC (2011). HIF1alpha and HIF2alpha: sibling rivalry in hypoxic tumour growth and progression. Nat Rev Cancer.

[6] Frost J (2016). Potent and selective chemical probe of hypoxic signalling downstream of HIF-alpha hydroxylation via VHL inhibition. Nat Commun.

[7] Martinez-Saez O, Gajate Borau P, Alonso-Gordoa T, Molina-Cerrillo J, Grande E (2017). Targeting HIF-2 alpha in clear cell renal cell carcinoma: A promising therapeutic strategy. Crit Rev Oncol Hematol.

[8] Zhao Z (2016). Synergy between von Hippel-Lindau and P53 contributes to chemosensitivity of clear cell renal cell carcinoma. Mol Med Rep.

[9] Zhang P (2013). Tubulin cofactor A functions as a novel positive regulator of ccRCC progression, invasion and metastasis. Int J Cancer.

[10] Ji SQ (2014). Down-regulation of CD74 inhibits growth and invasion in clear cell renal cell carcinoma through HIF-1alpha pathway. Urol Oncol.

[11] Motzer RJ (2007). Sunitinib versus interferon alfa in metastatic renal-cell carcinoma. N Engl J Med.

[12] Zhu J (2017). MEIS1 inhibits clear cell renal cell carcinoma cells proliferation and *in vitro* invasion or migration. BMC Cancer.

[13] Gryp, T., Vanholder, R., Vaneechoutte, M. & Glorieux, G. p-Cresyl Sulfate. Toxins **9**, 10.3390/toxins9020052 (2017). 10.3390/toxins9020052 PMC5331431 28146081

[14] Beaumont M (2017). Quantity and source of dietary protein influence metabolite production by gut microbiota and rectal mucosa gene expression: a randomized, parallel, double-blind trial in overweight humans. The American journal of clinical nutrition.

[15] Vanholder R, Schepers E, Pletinck A, Nagler EV, Glorieux G (2014). The uremic toxicity of indoxyl sulfate and p-cresyl sulfate: a systematic review. J Am Soc Nephrol.

[16] Jourde-Chiche N, Dou L, Cerini C, Dignat-George F, Brunet P (2011). Vascular incompetence in dialysis patients–protein-bound uremic toxins and endothelial dysfunction. Semin Dial.

[17] Watanabe H (2013). Molecular mechanisms for uremic toxin-induced oxidative tissue damage via a cardiovascular-renal connection. Yakugaku Zasshi.

[18] Woldu SL (2014). Renal insufficiency is associated with an increased risk of papillary renal cell carcinoma histology. Int Urol Nephrol.

[19] Satasivam P (2015). Patients with medical risk factors for chronic kidney disease are at increased risk of renal impairment despite the use of nephron-sparing surgery. BJU Int.

[20] Han H (2016). p-Cresyl sulfate promotes the formation of atherosclerotic lesions and induces plaque instability by targeting vascular smooth muscle cells. Front Med.

[21] Sun CY, Chang SC, Wu MS (2012). Uremic toxins induce kidney fibrosis by activating intrarenal renin-angiotensin-aldosterone system associated epithelial-to-mesenchymal transition. PloS one.

[22] Hwang, S. *et al*. Hypoxia-inducible factor-1alpha activates insig-2 transcription for degradation of HMG CoA reductase in the liver. *J Biol Chem*, 10.1074/jbc.M117.788562 (2017). 10.1074/jbc.M117.788562 PMC5454117 28416613

[23] Polke M (2017). Hypoxia and the hypoxia-regulated transcription factor HIF-1alpha suppress the host defence of airway epithelial cells. Innate Immun.

[24] Stoyanoff TR (2016). Tumor biology of non-metastatic stages of clear cell renal cell carcinoma; overexpression of stearoyl desaturase-1, EPO/EPO-R system and hypoxia-related proteins. Tumour Biol.

[25] Xi H (2014). Hypoxia inducible factor-1alpha suppresses Peroxiredoxin 3 expression to promote proliferation of CCRCC cells. FEBS Lett.

[26] Wang M (2017). AHNAK2 is a Novel Prognostic Marker and Oncogenic Protein for Clear Cell Renal Cell Carcinoma. Theranostics.

[27] Sun S (2009). Hypoxia-inducible factor-1alpha induces Twist expression in tubular epithelial cells subjected to hypoxia, leading to epithelial-to-mesenchymal transition. Kidney Int.

[28] Lee, H. M., Hwang, K. A. & Choi, K. C. Diverse pathways of epithelial mesenchymal transition related with cancer progression and metastasis and potential effects of endocrine disrupting chemicals on epithelial mesenchymal transition process. *Mol Cell Endocrinol*, 10.1016/j.mce.2016.12.026 (2016). 10.1016/j.mce.2016.12.026 28042023

[29] Zhang P (2014). Epithelial-mesenchymal transition is necessary for acquired resistance to cisplatin and increases the metastatic potential of nasopharyngeal carcinoma cells. Int J Mol Med.

[30] Song T (2011). MiR-138 suppresses expression of hypoxia-inducible factor 1alpha (HIF-1alpha) in clear cell renal cell carcinoma 786-O cells. Asian Pac J Cancer Prev.

[31] Chen YC, Chien LH, Huang BM, Chia YC, Chiu HF (2016). Aqueous Extracts of Toona sinensis Leaves Inhibit Renal Carcinoma Cell Growth and Migration Through JAK2/stat3, Akt, MEK/ERK, and mTOR/HIF-2alpha Pathways. Nutr Cancer.

[32] Bartel DP (2004). MicroRNAs: genomics, biogenesis, mechanism, and function. Cell.

[33] Bartel DP (2009). MicroRNAs: target recognition and regulatory functions. Cell.

[34] Medina PP, Slack FJ (2008). MicroRNAs and cancer: an overview. Cell Cycle.

[35] Li X (2014). MicroRNA-21 (miR-21) post-transcriptionally downregulates tumor suppressor PDCD4 and promotes cell transformation, proliferation, and metastasis in renal cell carcinoma. Cell Physiol Biochem.

[36] Cao J (2016). MicroRNA-21 stimulates epithelial-to-mesenchymal transition and tumorigenesis in clear cell renal cells. Mol Med Rep.

[37] Liu LZ (2011). MiR-21 induced angiogenesis through AKT and ERK activation and HIF-1alpha expression. PloS one.

[38] Song L (2016). MiR-21 modulates radiosensitivity of cervical cancer through inhibiting autophagy via the PTEN/Akt/HIF-1alpha feedback loop and the Akt-mTOR signaling pathway. Tumour Biol.

[39] Zhou Y (2016). Human Stem Cells Overexpressing miR-21 Promote Angiogenesis in Critical Limb Ischemia by Targeting CHIP to Enhance HIF-1alpha Activity. Stem Cells.

[40] Rossi M (2014). Uraemic toxins and cardiovascular disease across the chronic kidney disease spectrum: an observational study. Nutr Metab Cardiovasc Dis.

[41] Lin CJ (2014). P-cresyl sulfate is a valuable predictor of clinical outcomes in pre-ESRD patients. Biomed Res Int.

[42] Inoue T (2012). Genomic profiling of renal cell carcinoma in patients with end-stage renal disease. Cancer Sci.

[43] Shi SS (2013). Clear cell papillary renal cell carcinoma: a clinicopathological study emphasizing ultrastructural features and cytogenetic heterogeneity. Int J Clin Exp Pathol.

[44] Yamamoto S (2015). Continuous Reduction of Protein-Bound Uraemic Toxins with Improved Oxidative Stress by Using the Oral Charcoal Adsorbent AST-120 in Haemodialysis Patients. Sci Rep.

[45] Lee CT (2014). Effects of AST-120 on blood concentrations of protein-bound uremic toxins and biomarkers of cardiovascular risk in chronic dialysis patients. Blood Purif.

[46] Dong A, Shen J, Zeng M, Campochiaro PA (2011). Vascular cell-adhesion molecule-1 plays a central role in the proangiogenic effects of oxidative stress. Proceedings of the National Academy of Sciences of the United States of America.

[47] Lamberti MJ (2017). Transcriptional activation of HIF-1 by a ROS-ERK axis underlies the resistance to photodynamic therapy. PloS one.

[48] Krick S (2005). Hypoxia-driven proliferation of human pulmonary artery fibroblasts: cross-talk between HIF-1alpha and an autocrine angiotensin system. FASEB journal: official publication of the Federation of American Societies for Experimental Biology.

[49] Covello KL (2006). HIF-2alpha regulates Oct-4: effects of hypoxia on stem cell function, embryonic development, and tumor growth. Genes Dev.

[50] Schonenberger D (2016). Formation of Renal Cysts and Tumors in Vhl/Trp53-Deficient Mice Requires HIF1alpha and HIF2alpha. Cancer Res.

[51] Biswas S (2010). Effects of HIF-1alpha and HIF2alpha on Growth and Metabolism of Clear-Cell Renal Cell Carcinoma 786-0 Xenografts. J Oncol.

[52] Doonachar A (2015). Differential effects of HIF-alpha isoforms on apoptosis in renal carcinoma cell lines. Cancer Cell Int.

[53] Fan Y (2016). Dicer suppresses the malignant phenotype in VHL-deficient clear cell renal cell carcinoma by inhibiting HIF-2alpha. Oncotarget.

[54] Xu J (2012). Epigenetic regulation of HIF-1alpha in renal cancer cells involves HIF-1alpha/2alpha binding to a reverse hypoxia-response element. Oncogene.

[55] Raval RR (2005). Contrasting properties of hypoxia-inducible factor 1 (HIF-1) and HIF-2 in von Hippel-Lindau-associated renal cell carcinoma. Mol Cell Biol.

[56] Hicks KC, Patel TB (2016). Sprouty2 Protein Regulates Hypoxia-inducible Factor-alpha (HIFalpha) Protein Levels and Transcription of HIFalpha-responsive Genes. J Biol Chem.

[57] Ding XF, Zhou J, Hu QY, Liu SC, Chen G (2015). The tumor suppressor pVHL down-regulates never-in-mitosis A-related kinase 8 via hypoxia-inducible factors to maintain cilia in human renal cancer cells. J Biol Chem.

[58] Chen L (2016). Physapubescin selectively induces apoptosis in VHL-null renal cell carcinoma cells through down-regulation of HIF-2alpha and inhibits tumor growth. Sci Rep.

[59] Schoenfeld AR (2000). The von Hippel-Lindau tumor suppressor gene protects cells from UV-mediated apoptosis. Oncogene.

[60] Zhang KL (2014). Blockage of a miR-21/EGFR regulatory feedback loop augments anti-EGFR therapy in glioblastomas. Cancer Lett.

