# Supplementary material for: The uremic toxin p-cresyl sulfate induces proliferation and migration of clear cell renal cell carcinoma via microRNA-21/ HIF-1α axis signals
Source: Sci Rep. 2019 Mar 1;9:3207. doi: 10.1038/s41598-019-39646-9 (PMC6397167; doi:10.1038/s41598-019-39646-9)
Supplement: Supplementary file 1 — Supplementary Information [file 41598_2019_39646_MOESM1_ESM.docx]

**The uremic toxin p-cresyl sulfate induces proliferation and migration of clear cell renal cell carcinoma via microRNA-21/ HIF-1α axis signals**

TSAI-KUN WU^1,2^, CHYOU-WEI WEI^3,4^, YING-RU PAN^2,3^, REN-JUN HSU^5^, CHUNG-YI WU^1,6^, and YUNG-LUEN YU^1,7,8,9,10 *^

^1^The Ph.D. Program for Cancer Biology and Drug Discovery, China Medical University and Academia Sinica, Taichung 404, Taiwan.

^2^Division of Renal Medicine, Tungs' Taichung Metroharbor Hospital, Taichung 435, Taiwan.

^3^Deparment of Nutrition, Master Program of Biomedical Nutrition, Hungkuang University, Taichung 433, Taiwan.

^4^Department of Nursing, Hungkuang University, Taichung 433, Taiwan.

^5^Graduate Institute of Life Sciences, National Defense Medical Center, Taipei 114, Taiwan.

^6^The Genomics Research Center, Academia Sinica, Taipei 115, Taiwan

^7^Graduate Institute of Biomedical Sciences, China Medical University, Taichung 404, Taiwan.

^8^Drug Development Center, China Medical University, Taichung 404, Taiwan.

^9^Center for Molecular Medicine, China Medical University Hospital, Taichung 404, Taiwan.

^10^Department of Biotechnology, Asia University, Taichung 413, Taiwan.

***Correspondence to:**

**Dr. Yung-Luen Yu, Graduate Institute of Biomedical Sciences, China Medical University, 6 Hsueh Shih Road, Taichung 404, Taiwan. E mail: ylyu@mail.cmu.edu.tw**

** all lane in our studies run together on the original gel / bands, so we do not insert any black line between lanes

** Red line in each picture indicated the cropped image in the figures.

**SUPPLEMENTARY INFORMATION FILE**

**Supplementary Figure S1.** Original images for effect of PCS on HIF-1α, HIF-2α and VHL levels of 786-O and A498 cells, treated with 100 or 200μM PCS for 1-5 day The manipulated versions are reported in Fig. 2 of the manuscript.

Fig.2(a)-1 blot image of HIF-1α (dot line) Fig.2(b)-1 blot image of HIF-1α (dash line)







Long exposure of Fig.2(a)-1 Long exposure of Fig.2(b)-1







Fig.2(a)-2 blot image of HIF-2α (dot line) Fig.2(b)-2 blot image of HIF-2α (dash line)







Long exposure of Fig.2(a)- 2 Long exposure of Fig.2(b)-2







Fig.2(a)-3 blot image of VHL (dot line) Fig.2(b)-3 blot image of VHL (dash line)







Long exposure of Fig.2(a)-3 Long exposure of Fig.2(b)-3







Fig.2(a)-4 blot image of α-Tubulin (dot line) Fig.2(b)-4 blot image of α-Tubulin (dash line)







Long exposure of Fig.2(a)-4 Long exposure of Fig.2(b)-4







**Supplementary Figure S2.** Original images for effect of PCS on the expression of EMT-related proteins in 786-O and A498 cells. The manipulated versions are reported in Fig. 3 of the manuscript.

Fig.3(a)-1 blot image of Fibronectin (dot line) Fig.3(b)-1 blot image of Fibronectin (dash line)







Long exposure of Fig.3(a)-1 Long exposure of Fig.3(b)-1







Fig.3(a)-2 blot image of E-cadherin (dot line) Fig.3(b)-2 blot image of E-Cadherin (dash line)







Long exposure of Fig.3(a)-2 Long exposure of Fig.3(b)-2







Fig.3(a)-3 blot image of Twist (dot line) Fig.3(b)-3 blot image of Twist (dash line)







Fig.3(a)-4 blot image of Vimentin (dot line) Fig.3(b)-4 blot image of Vimentin (dash line)







Long exposure of Fig.3(a)-4 Long exposure of Fig.3(b)-4







Fig.3(a)-5 blot image of α-Tubulin (dot line) Fig.3(b)-5 blot image of α-Tubulin (dash line)







Long exposure of Fig.3(a)-2 Long exposure of Fig.3(b)-2







**Supplementary Figure S3.** Original images for effect of HIF-1α knockdown on HIF-1α, HIF-2α, and VHL expressions. HIF-1α, HIF-2α, VHL and α-tubulin levels were assayed by western blotting. The manipulated versions are reported in Fig. 4 of the manuscript.

Fig.4(a)-1 blot image of HIF-1α (dot line) Fig.4(a)-2 blot image of HIF-2α (dash line)







Fig.4(a)-3 blot image of VHL (dot line) Fig.4(a)-4 blot image of α-Tubulin (dash line)







Long exposure of Fig.4(a)-3 Long exposure of Fig.4(a)-





**Supplementary Figure S4.** Original images for effect of HIF-1α knockdown on expression of EMT-related proteins assayed by western blotting. The manipulated versions are reported in Fig. 5 of the manuscript.

Fig.5(a)-1 blot image of Fibronectin (dot line) Fig.5(a)-2 blot image of E-Cadherin (dash line)







Fig.5(a)-3 blot image of Twist (dot line) Fig.5(a)-4 blot image of Vimentin (dash line)





Long exposure of Fig.5(a)-3 Long exposure of Fig.5(a)-4







Fig.5(a)-5 blot image of α-Tubulin (dot line)





**Supplementary Figure S5.** Original images for effect of miR-21 inhibitor on PCS-induced HIF-1α mRNA expression by PCR and HIF-1α, HIF2α and VHL expressions by western blotting. The manipulated versions are reported in Fig. 6 of the manuscript.

Fig.6(b)-1 blot image of HIF-1α (dot line) Fig.6(b)-2 full blot image of HIF-2α (dash line)







Fig.6(b)-3 full blot image of VHL (dot line) Fig.6(b)-4 blot image of α-Tubulin (dash line)







**Supplementary Figure S6.** Original images for effect of miR-21 inhibitor on PCS-induced expression of EMT-related proteins by western blotting. The manipulated versions are reported in Fig. 6 of the manuscript.

Fig.6(f)-1 blot image of Fibronectin (dot line) Fig.6(f)-2 blot image of E-Cadherin (dash line)







Long exposure of Fig.6(f)-1 Long exposure of Fig.6(f)-2







Fig.6(f)-3 blot image of Vimentin (dot line) Fig.6(f)-4 blot image of Twist (dash line)







Long exposure of Fig.6(f)-3





Fig.6(f)-5 blot image of α-Tubulin





Long exposure of Fig.6(f)-5
